# Transmission of *Beauveria bassiana *from male to female *Aedes aegypti *mosquitoes

**DOI:** 10.1186/1756-3305-4-24

**Published:** 2011-02-26

**Authors:** Alberto M García-Munguía, Javier A Garza-Hernández, Eduardo A Rebollar-Tellez, Mario A Rodríguez-Pérez, Filiberto Reyes-Villanueva

**Affiliations:** 1 Laboratorio de Entomología Médica, Facultad de Ciencias Biológicas, Universidad Autónoma de Nuevo León, Pedro de Alba S/N Ciudad Universitaria, Apdo. Postal 109-F, 66450, San Nicolás de los Garza, Nuevo León, México; 2 Laboratorio de Biomedicina Molecular, Centro de Biotecnología Genómica, Instituto Politécnico Nacional, Boulevard del Maestro S/N esquina Elías Piña. Col. Narciso Mendoza, 88710, Cd. Reynosa, Tamaulipas, México

## Abstract

**Background:**

Resistance to chemical insecticides plus high morbidity rates have lead to rising interest in fungi as candidates for biocontrol agents of mosquito vectors. In most studies fungal infections have been induced by exposure of mosquitoes to various surfaces treated with conidia. In the present study eight Mexican strains of *Beauveria bassiana *were assessed against *Aedes aegypti *by direct exposure of females to 6 × 10^8 ^conidia ml ^-1 ^on a filter paper, afterwards, the transmission of the least and most virulent isolates was evaluated by mating behavior from virgin, fungus-contaminated male to females, to examine this ethological pattern as a new approach to deliver conidia against the dengue vector.

**Methods:**

In an exposure chamber with a filter paper impregnated with 6 × 10^8 ^conidia ml ^-1 ^of the least and most virulent strains of *B. bassiana*, 6-8 day old males of *A. aegypti *were exposed for 48 hours, and then transferred individually (each one was a replicate) to another chamber and confined with twenty healthy females of the same age. Clean males were used in controls. Survival, infection by true mating (insemination) or by mating attempts (no insemination) and fecundity were daily registered until the death of last female. Data analysis was conducted with proc glm for unbalanced experiments and means were separated with the Ryan test with SAS.

**Results:**

All strains were highly virulent with LT_50 _ranging from 2.70 (± 0.29) to 5.33 (± 0.53) days. However the most (Bb-CBG2) and least virulent (Bb-CBG4) isolates were also transmitted by mating behavior; both killed 78-90% of females in 15 days after being confined with males that had previously been exposed for 48 hours to fungi. Of these mortality rates, 23 and 38% respectively, were infections acquired by copulations where insemination occurred. The LT_50 _for sexually-infected females were 7.92 (± 0.46) and 8.82 (± 0.45) days for both strains, while the one in control was 13.92 (± 0.58). Likewise, fecundity decreased by 95% and 60% for both Bb-CBG2 and Bb-CBG4 isolates in comparison with control. The role of mating attempts in this delivery procedure of *B. bassiana *is discussed.

**Conclusions:**

This is the first report about transmission of *B. bassiana *by mating behavior from virgin, fungus-contaminated males to females in *A. aegypti*. Fungal infections acquired by this route (autodissemination) infringed high mortality rates (90%) in mated or approached females. However, prior to releasing virgin, fungus-contaminated males to spread *B. basasiana *among females of *A. aegypti*, this novel alternative needs further investigations.

## Background

*Beauveria bassiana *is a soil-borne cosmopolitan fungus that infects mostly soil-dwelling insects [1]. For forty two years it has been known that mosquito adults of *Culex*, *Anopheles albimanus*, and the dengue vector *Aedes aegypti *are susceptible to infections by this pathogen [2]. Recently, this fungus and *Metarhizium anisopliae *as well have received considerable attention by medical entomologists as potential microbial control agents against the malaria [3-5]) and dengue vectors [6,7]. The mortality of adult mosquitoes has been evaluated in many studies after various methods of fungal infection involving both dry and oil-formulated conidia as appears in a recent review [8]). Nonetheless, these fungi could be also disseminated by virgin males toward females in the case of the dengue vector *A. aegypti *due to the male tendency to mate multiple times with different females [9]. An early report stated that a virgin male of *A. aegypti *is capable of mating with and inseminating up to seven females after the first thirty minutes of confinement in a cage [10]. Besides, preliminary observations on the sexual activity of *A. aegypti *virgin males in our laboratory showed that a 6-8 day old male confined with 20 females of the same age inseminated an average of 14, 13 and 5 females after the first 0.5, 1 and 24 hours of captivity in cages (one for each time) (unpublished data). Fungal transmission by sexual activity in insects is a type of horizontal transmission known as autodissemination because occurs between individuals of the same species and generation [11]. To our knowledge, the first report of this type of transmission in vectors of human diseases was for *M. anisopliae *in the tsetse fly *Glossina morsitans morsitans *in 1990 [12]. Following this, previous work in our laboratory has shown that *M. anisopliae *was transmitted by real mating (females infected and inseminated) or copulation attempts (females infected but non-inseminated) from virgin *A. aegypti *males inoculated with conidia to *A. aegypti *females (unpublished data); we demonstrated that a highly virulent strain of *M. anisopliae *caused 90% mortality plus an effect of sterilization when fecundity was recorded in infected females. Therefore, in the present study we evaluated: 1) the virulence of eight Mexican strains of *B. bassiana*, after passage through mosquito adults, against females of *A. aegypti *by exposure of insects to filter papers impregnated with conidia, 2) the transmission rate by mating behavior (true mating and mating attempts) from virgin males to females for two isolates, and 3) the impact of both strains transmitted by sexual activity upon female fecundity.

## Methods

### Mosquitoes

A colony of *Aedes aegypti *was established with larvae collected from dengue endemic neighborhoods located at Monterrey, NL, Mexico. Male and female mosquitoes emerging within a 24 h period were kept together for mating and were provided with cotton pads soaked in 5% sucrose solution *ad libitum*. Adults were maintained at 27 (± 2)°C, 85 (± 10%) RH in a 12:12 h L:D photoperiod. Insects were blood fed on the forearm of one of the authors (AMGM) to stimulate egg production. Following the blood meal, oviposition occurred in beakers half filled with water and lined with filter paper. Egg eclosion was stimulated by total immersion of the filter paper in water at 37°C but previously boiled to reduce oxygen tension, in which 0.04 grams of "Tetramin^®^" was added for neonates. Larvae were maintained at a density of 200/liter in plastic trays and fed with 3 grams of the same food during the 2^nd ^and 5^th ^day. Pupae were switched into water-filled beakers and transferred to cages for adult emergence. Recently hatched males and females (3-day old) were separated for bioassays.

### Fungal strains and preparation of conidia suspension

Eight strains of *B. bassiana *described in Table 1 were collected from different localities (States) in Mexico. All were cultured first on potato-dextrose-agar (PDA) and incubated at 25°C for 20 days for conidiation. Following incubation, conidia harvest was prepared in 0.5% Tween 20 in 0.85% sodium chloride in distilled water (5 ml of Tween 20 in 1 liter of saline solution) from plates using a micro spatula to carefully separate the spore layer from the agar. Later, a small number of *A. aegypti *females were infected with *B. bassiana *and the pathogen re-isolated from the sporulating cadavers by removing a sample of conidia from the exterior of the cadaver and inoculating again on PDA in Petri dishes. These isolation plates were incubated at 25°C for 20 days. Then, conidia from uncontaminated plates were used to prepare a concentration of 6 × 10^8 ^conidia ml^-1 ^per isolate, determined using a Fisher hemocytometer.

**Table 1:** Median Lethal Time (LT_50_)^1 ^± Standard Error (SE) in days computed for samples of forty 6-8 day old females of *A. aegypti *after exposure for 48 hours to a filter paper impregnated with 6 × 10^8 ^conidia ml ^-^^1 ^of each one of eight isolates of *B. bassiana *collected from various localities in Mexico

Isolate^1^	LT_50 _± SE	Host/Source	Locality and State
Bb-CBG1	5.03 ± 0.69	Soil	Texcoco, México.
Bb-CBG2	2.70 ± 0.29*	Coleoptera (*Aphodius *sp.)	Zuazua, Nuevo León
Bb-CBG3	3.20 ± 0.42	Soil	Marín, Nuevo León
Bb-CBG4	5.33 ± 0.53*	Soil	Metztitlan, Hidalgo
Bb-CBG5	4.06 ± 0.46	Soil	Marín, Nuevo León
Bb-CBG6	3.30 ± 0.47	Soil	Cuernavaca, Morelos
Bb-CBG7	3.46 ± 0.47	Soil	La Ceiba, Puebla
Bb-CBG8	3.60 ± 0.51	Soil	Chapingo, México.
Control	14.26 ± 0.43		

^1 ^Significance within the same column, χ^2 ^= 194.85, df = 8, p < 0.0001.

*Isolates with the highest and lowest virulence.

### Infection of mosquitoes

Two bioassays were conducted to study the effect of: 1) conidia of eight strains of *B. bassiana *on survival of adult female *A. aegypti*. In this bioassay 1, exposure of females to conidia was for 48 hours to estimate the virulence as the median lethal time (LT_50_) for each strain. 2) limited exposure (48 hours) of females to virgin males previously inoculated for 48 hours with conidia of two fungal strains (those found to be the most and least virulent in the results of bioassay 1 to evaluate the impact of conidia transmitted by mating behavior on female survival, infection (inseminated and not), mortality and fecundity.

**Figure 1:**
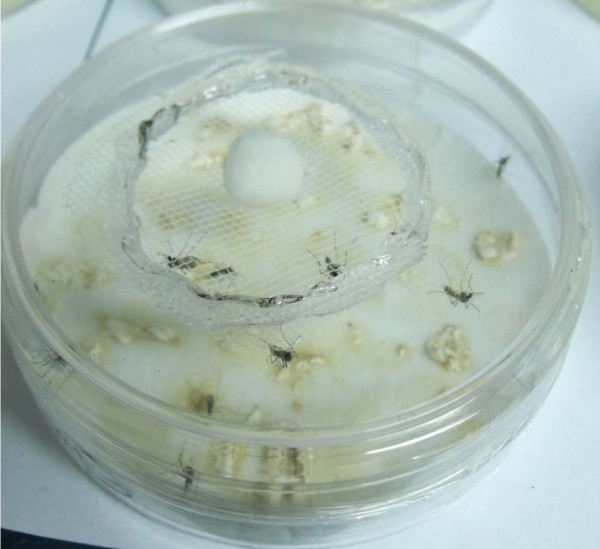
**Chamber for exposure of *A. aegypti *to 6 × 10^8 ^conidia ml^-1 ^of *B. bassiana***. A cotton ball soaked with 5% sucrose was placed over the net of the hole at top half.

In bioassay 1, nine treatments were set up: the eight strains of *B. bassiana *plus a control (filter paper only with solutions without fungus). Each treatment was twice replicated, and twenty females were tested per replicate. To prepare one treatment, seven ml with a concentration of 6 × 10^8 ^conidia ml^-1 ^was poured on a sterile filter paper in a Petri dish and allowed to dry at 25°C, 60% RH, in laboratory for 24 hours before being placed into an exposure chamber (Figure 1) constructed by two half dishes positioned upside down, and both halves taped. A 1 cm hole covered with net in the top half allowed the introduction of twenty 6-8 day old female mosquitoes with a mouth aspirator. Following a 48-h period, two groups of twenty females each were separated; each group was removed from the chamber and switched to 1-liter plastic pot with a cotton mesh-netting top. Pots were maintained at laboratory conditions described above for filter drying. Insects were fed on 5% sucrose offered on cotton pads placed on the netting surface of each pot. Dead insects were removed daily and rinsed in 1% sodium hypochlorite for 20 seconds and then washed twice in distilled water for 20 seconds. All dead mosquitoes were placed in Petri dishes lined with damp filter paper and maintained at 25°C to stimulate conidiogenesis.

For bioassay 2, three treatments were prepared. Two isolates: the most (Bb-CBG2) and the least virulent (Bb-CBG4) that resulted from the bioassay 1, plus the control; each one was twice replicated, twenty females per replicate. Ten 6-8 day old virgin males were exposed for 48 hours to the same dose for each strain. Thereby, four contaminated males (two of each strain) were transferred individually to 1-liter plastic pots with a cotton mesh-netting top, and confined with twenty 6-8 day old females. Clean males were introduced with twenty females each in two plastic pots as a control. Insects of each replicate were confined for just 48 hours with a male, and blood fed on the forearm of the same volunteer (AMGM) in the first six hours of confinement. Afterwards, engorged females were transferred individually to beakers half filled with water and lined with filter paper for oviposition. All females were dissected immediately after death to check for the presence of sperm in the spermathecae and retention of fully developed eggs in ovaries. Fecundity was considered as the sum of laid and retained eggs from the first gonotrophic cycle. After dissection the cadavers were immersed in 1% sodium hypochlorite for 20 seconds and then washed twice in distilled water for 20 seconds. All carcasses were placed in a humid Petri dish chamber for sporulation to confirm death by the fungus. Mortality and infection rate by successful (insemination) and failed (no insemination) pairings were evaluated on a daily basis until death of the last female in both treated groups and control.

### Statistical analyses

The median lethal time (LT_50_) was obtained from the survival analysis computed with the Kaplan-Meier model for the forty females per treatment in both bioassays. Each curve was computed by pooling the two replicates per treatment, after previously performing a test for variation between both replicates by analysis of variance (ANOVA). The mortality and infection rates for true mating (females inseminated and then sporulated), mating attempt (females non-inseminated and then sporulated), and mean fecundity among treatments were analyzed by ANOVA for unbalanced experiments, and Ryan tests for multiple mean comparisons were also computed with proc glm in SAS [13].

## Results

### Susceptibility of *A. aegypti *females to eight strains of *B. bassiana*

The results shown in Table 1 demonstrated that all fungal strains caused significantly increased mortality (χ^2 ^= 194.85, df = 8, p < 0.0001). The 50% mortality (LT_50_) was reached in all strains within the first five days after of the initial exposure to the fungi, whereas in the control the LT_50 _was 14 days after of fungal exposure, therefore the maximum life of treated mosquitoes was around 11 days, while those in the control lived almost 25 days. In this screening assay the mortality was evaluated by exposure to filter papers impregnated with 6 × 10^8 ^conidia ml ^-1 ^and allowed the identification of the most (Bb-CBG2) and the least virulent (Bb-CBG4) isolates, which had an LT_50 _of 2.70 (± 0.29) and 5.33 (± 0.53) days respectively.

### Susceptibility of *A. aegypti females *to two *B. bassiana *strains transmitted by sexual behavior

**Figure 2:**
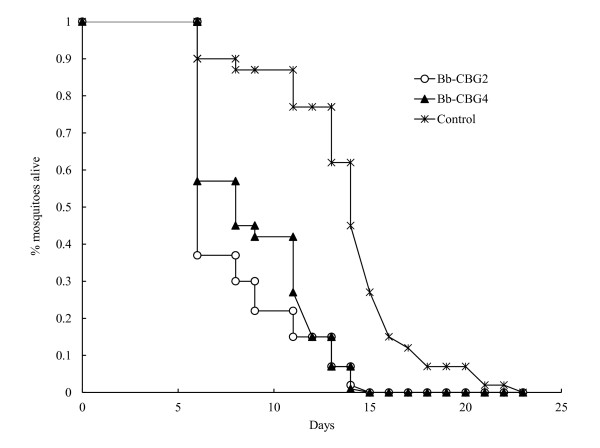
**Mean cumulative proportional survival (±Standard Error) calculated by the Kaplan-Meier model for forty females of *A. aegypti *confined with a virgin male previously exposed to 6 × 10^8 ^conidia ml^-1 ^of two isolates of *B. bassiana *plus Control (healthy male)**. Mortality by fungus was demonstrated by sporulation in cadavers (see text).

**Figure 3:**
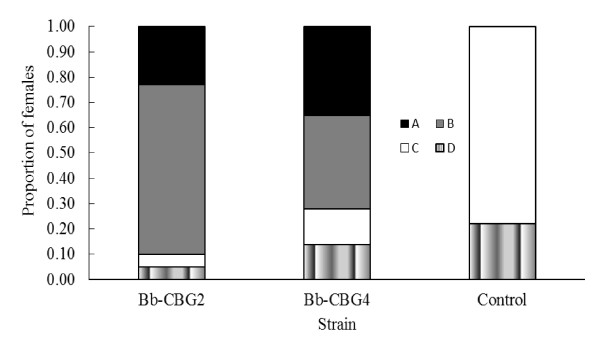
**Proportion of four categories of females of *A. aegypti *confined with a fungus-contaminated male, for two strains of *B. bassiana *plus control (clean male)**. Symbols: A = Sporulated-inseminated, B = Sporulated-not inseminated, C = Not sporulated-inseminated, D = Not sporulated-not inseminated females.

Results of bioassay 2 that comprised these two strains and the control demonstrated the fungal transmission by mating behavior from contaminated males to healthy females. Figure 2 shows the survival curves for females confined with a male previously inoculated with either one of the isolates identified in bioassay 1 and the control. Each curve describes the daily death probability for all individuals per treatment, calculated by the Kaplan-Meier model. From these analyses the resulting LT_50 _were 7.92 (± 0.46), 8.82 (± 0.48), and 13.92 (± 0.58) days for the Bb-CBG2, Bb-CBG4, and the control, respectively (χ^2 ^= 56.29, df = 2, p < 0.0001). Overall, among the forty mosquitoes placed together with the male contaminated with the Bb-CBG2, there were 36 sporulating females (9 with eggs and 27 with no eggs) while in the non-sporulating ones, two laid eggs and other two did not lay eggs. For females confined with the male inoculated with the Bb-CBG4, 31 were mycosed (15 with eggs and 16 with no eggs) and 9 non-mycosed, of which three laid eggs and six did not lay eggs. The females whose carcasses showed conidiogenesis but laid eggs before death represent the females killed by the fungal infection acquired by successful mating where insemination occurred. While those that sporulated without laying eggs comprised the sector of females killed by the pathogen but where infections were transmitted through copulation attempts or other physical contacts carried out by the contaminated male. Figure 3 shows the results of bioassay 2 where both Bb-CBG2 and Bb-CBG4 caused the same total 78-90% (31, 36/40) mortality (mosquitoes with conidiation) in females exposed to a male contaminated with either isolate, however, this mortality was registered at the end of 15 days, an interval shorter than 22 days that the healthy mosquitoes lived in the control (F = 157.39, df = 2, p < 0.0001). According to data, 23 and 38% of mosquitoes killed by the fungi actually were infected through copulations with successful inseminations (true matings). The rates of mortality for females infected but not inseminated were 67 (27/40) and 40% (16/40) representing likely the mortality of failed copulations (no insemination). The insemination rate in the control was 78%.

Lastly, both strains of *B. bassiana *transmitted by mating behavior exerted a negative impact on egg production. Fecundity of females exposed to the male with the virulent strain Bb-CBG2 had a mean of 2.05 (± 1.02) eggs per female; this mean was 95% lower than the one observed for healthy females in the control which was 42.56 (± 6.90). Likewise, the less virulent Bb-CBG4 diminished the fecundity in 67% in comparison with the control (F = 165.30, df = 3, p < 0.0001). The secondary pathogenic effect also was observed in the number of ovipositing females (with sporulation) per treatment because these were 9 and 15 for the Bb-CBG2 and Bb-CBG4, and 31 in the control. Besides, in both isolates there were non-infected females that laid eggs. In general, 4 and 9 out of 40 females were not infected (without conidiogenesis), and of these, 2 and 3 laid eggs before death.

## Discussion

This is the first study that demonstrates the transmission of *B. bassiana *by mating behavior from virgin males contaminated with conidia to healthy females in *A. aegypti*. Concerning the bioassay 1, the range 2.70 (± 0.29) - 5.33 (± 0.53) days of the LT_50 _observed in Table 1 for the eight isolates of *B. bassiana *we tested against *A. aegypti*, is similar to the 4.1 (± 0.3) days observed for an African strain of *Metarhizium anisopliae *[6]. However, our strains were more pathogenic than the ones investigated in Brazil [7] where assessed three isolates of *B. bassiana *against *A. aegypti *females by indirect contact to a fungal suspension of 1 × 10^9 ^conidia ml^-1^; they reported a mean total mortality of 26, 30 and 70% for the three strains, while the LT_50 _was estimated in 4 days only for the most virulent isolate. Nevertheless, they did not pass the strains through mosquito adults and then re-isolated the fungi on PDA to be used in their bioassays. Other relevant point in bioassay 1 is that the most virulent strain Bb-CBG2 was the only one isolated originally from a dead insect with sporulation, while the rest were isolated from soil (Table 1). Of particular relevance for our study is the number of females that were infected by mating behavior but not inseminated, because they could represent an indirect measure of the transmission of *B. bassiana *by copulation attempts; moreover, also is the chance of acquiring conidia from plastic pots and this possibility is the same for both inseminated and not inseminated females, and we did not evaluate the transmission rate through this path. Unfortunately there is not a study reporting accurate measures of successful and failed matings in virgin males of *A. aegytpi *to discuss this point. There is just a recent paper [14] where the insemination rate was determined in females confined with virgin males, but in healthy insects, and the rate varied from 69 to 89%, a range similar to the one (80-90%) of our study. It is important to mention that the males inoculated with the virulent Bb-CBG2 died within the 4-6 days after fungus exposure while those with the Bb-CBG4 died in 5-8 days. Whether the males are capable of detecting a severe pathogenic process and then switching to a more aggressive sexual behavior, still remains unknown; however a male infected with a virulent strain is invaded more rapidly by the fungus, and paralysis produced by dextrusins is one of the pathogenic effects [15]. Perhaps the males infected with the Bb-CBG2 were incapable of inseminating the majority of females they approached due to their weakness and slow movements or flight, although the females could also refuse to mate with sick males. Further investigations are necessary to determine the impact of virulence on the role of copulation attempts in this type of horizontal transmission of fungi in *A. aegypti*.

There are only two reports that address the transmission of fungi among mosquitoes during mating, but their results are not comparable with our study because of the different methodologies. In one study [3] found only 34.0% mortality in males after pairing inoculated females of the mosquito *Anopheles gambiae *(s. s.) during a confinement of 1 hour, where clean females were previously exposed for 24 hours to 1.6 × 10^10 ^conidia m^-2 ^of an isolate of *M. anisopliae*, a study quite different to ours; we inoculated males instead of females, and we exposed them for 48 hours to *B. bassiana*. The second report was our first study (unpublished data) in this research line but with two Mexican isolates of *M. anisopliae*; we found 90% mortality in females exposed to a male contaminated with the strain CBG-Ma-2 applied with the same method we used here. In addition, a paper stated for the tsetse fly, *Glossina morsitans morsitans *[12] reporting a 55.0% mortality of clean females paired for two weeks with males that were sprayed with a fungal suspension either of *M. anisopliae *or *B. bassiana*.

Reduction in fecundity is a secondary effect of fungal infections in insects; moreover there is little data for mosquitos. To our knowledge, the first report was in 1985 [16] where observed a reduction in egg viability of *A. aegypti *mosquitoes infected with the entomopathogenic fungus, *Aspergillus parasiticus *Speare. In the other case [17] infected *A. gambiae *adults with *M. anisopliae *but not by mating transmission; anyway they observed that the decrease in egg-laying capacity was most likely to be a direct effect of the reduced amount of blood ingested per blood meal. Finally is our early report (unpublished data) about *M. anisopliae *transmitted by mating behavior, where we observed a severe decrease in the mean fecundity to almost zero (sterilization) in infected females of *A. aegypti*, compared with fecundity in control.

Results of studies about the impact of entomopathogenic fungi on human health are controversial. Although they causing opportunistic infections in man [18], they also offer a given extent of safety in human habitats [19]. Whether the intention of our research line is to releasing virgin, fungus-contaminated males of *A. aegypti *at outdoor and indoor conditions to establish a dengue biocontrol, the males will spread conidia not only on females by mating but everywhere each time they make contact with any surface, including skin or head hair of humans. The amount of conidia a male is capable of carrying is unknown; this issue and others are part of ongoing investigations in semi-field and field conditions to explore in more detail this delivery procedure of *B. bassiana *from males to females of *A. aegypti *in Mexico.

## Conclusions

Eight Mexican strains of *B. bassiana *(after a mosquito-passage) were highly pathogenic against *A. aegypti *females with a maximum LT_50 _of five days, by exposure of insects to filter papers impregnated with 6 × 10^8 ^conidia ml ^-1^.

This is the first report of transmission of *B. bassiana *by mating behavior from virgin, fungus-contaminated males to females in *A. aegypti*, causing 90% mortality in 15 days. The strains Bb-CBG2 and Bb-CBG4 transmitted sexually from contaminated males decreased the fecundity in 95 and 67% in exposed females.

## Competing interests

The authors declare that they have no competing interests.

## Authors' contributions

AMGM collected the fungi in field and isolated them in laboratory, he also performed the bioassays. JAGH collected the *A. aegypti *in field and established the colony used in this study. He is also responsible for maintenance of experimental strains of *B. bassiana *in our laboratory. EART was responsible for the maintenance of mosquito colonies. MARP helped to conceive the original objective of the study, and participated in draft the MS. FRV is responsible for the original idea for this study, conceived the experimental design, performed the statistical analyses and prepared the early draft.
